# Supplementary material for: Daily feeding rhythm linked to microbiome composition in two zooplankton species
Source: PLoS One. 2022 Feb 3;17(2):e0263538. doi: 10.1371/journal.pone.0263538 (PMC8812976; doi:10.1371/journal.pone.0263538)
Supplement: S1 Table — The mean relative abundance in both host species is listed for each ASV as well as the taxonomic identity. (PDF) [file pone.0263538.s001.pdf]

Core to:	Mean relative abundance in *Daphnia magna*	Mean relative abundance in *Daphnia dentifera*	Taxonomic identity					
			Phylum	Class	Order	Family	Genus	Species
Both	0.512	0.121	Proteobacteria	Gammaproteobacteria	Burkholderiales	Burkholderiaceae	Limnohabitans	NA
Both	0.213	0.066	Bacteroidota	Bacteroidia	Chitinophagales	Chitinophagaceae	NA	NA
Both	0.051	0.128	Proteobacteria	Gammaproteobacteria	Burkholderiales	Burkholderiaceae	Hydromonas	Hydromonas_duriensis(RS_GCF_004363775.1)
Both	0.008	0.008	Bacteroidota	Bacteroidia	Chitinophagales	Saprospiraceae	NA	NA
Both	0.005	0.028	Bacteroidota	Bacteroidia	Cytophagales	Spirosomaceae	Emticicia	Emticicia_oligotrophica(GB_GCA_000263195.1)
Both	0.004	0.064	Bacteroidota	Bacteroidia	Flavobacteriales	Flavobacteriaceae	Flavobacterium	NA
Both	0.003	0.044	Proteobacteria	Gammaproteobacteria	Burkholderiales	Burkholderiaceae	Vitreoscilla_A	Vitreoscilla_A_sp004359425(GB_GCA_004359425.1)
Daphnia magna	0.068	0.000	Proteobacteria	Gammaproteobacteria	Burkholderiales	Burkholderiaceae	NA	NA
Daphnia magna	0.037	0.000	Bacteroidota	Bacteroidia	Cytophagales	Spirosomaceae	Arcticibacterium	Arcticibacterium_luteifluviistationis(RS_GCF_003258705.1)
Daphnia magna	0.029	0.001	Proteobacteria	Gammaproteobacteria	Pseudomonadales	NA	NA	NA
Daphnia magna	0.008	0.000	Bacteroidota	Bacteroidia	Chitinophagales	Chitinophagaceae	NA	NA
Daphnia magna	0.007	0.000	Bacteroidota	Bacteroidia	Cytophagales	Spirosomaceae	Emticicia	Emticicia_oligotrophica(GB_GCA_000263195.1)
Daphnia magna	0.004	0.000	Bacteroidota	Bacteroidia	Flavobacteriales	Flavobacteriaceae	Flavobacterium	Flavobacterium_sp004303025(RS_GCF_004303025.1)
Daphnia magna	0.004	0.000	Bacteroidota	Bacteroidia	Flavobacteriales	Crocinitomicaceae	Fluviicola	Fluviicola_taffensis(RS_GCF_000194605.1)
Daphnia magna	0.004	0.000	Bacteroidota	Bacteroidia	Chitinophagales	Chitinophagaceae	OLB11	OLB11_sp001567165(GB_GCA_001567165.1)
Daphnia magna	0.004	0.001	Myxococcota	Polyangia	Polyangiales	Polyangiaceae	NA	NA
Daphnia magna	0.003	0.000	Proteobacteria	Gammaproteobacteria	Burkholderiales	Burkholderiaceae	Aquabacterium	NA
Daphnia magna	0.002	0.000	Bacteroidota	Bacteroidia	Flavobacteriales	Flavobacteriaceae	Flavobacterium	NA
Daphnia magna	0.002	0.000	Proteobacteria	Gammaproteobacteria	Burkholderiales	Burkholderiaceae	NA	NA
Daphnia magna	0.001	0.000	Planctomycetota	Planctomycetes	Gemmatales	Gemmataceae	Fimbriiglobus	Fimbriiglobus_ruber(RS_GCF_002197845.1)
Daphnia dentifera	0.002	0.005	Proteobacteria	Gammaproteobacteria	Burkholderiales	Burkholderiaceae	Limnobacter	NA
Daphnia dentifera	0.001	0.097	Proteobacteria	Gammaproteobacteria	Burkholderiales	Burkholderiaceae	NA	NA
Daphnia dentifera	0.001	0.010	Proteobacteria	Gammaproteobacteria	Nevskiales	Nevskiaceae	Nevskia	Nevskia_ramosa(RS_GCF_000420645.1)
Daphnia dentifera	0.001	0.003	Bacteroidota	Bacteroidia	Cytophagales	Spirosomaceae	Runella	Runella_sp003339505(RS_GCF_003339505.1)
Daphnia dentifera	0.001	0.009	Proteobacteria	Gammaproteobacteria	Nevskiales	Nevskiaceae	Nevskia	Nevskia_ramosa(RS_GCF_000420645.1)
Daphnia dentifera	0.000	0.045	Actinobacteriota	Actinomycetia	Nanopelagicales	Nanopelagicaceae	Planktophila	NA
Daphnia dentifera	0.000	0.002	Proteobacteria	Gammaproteobacteria	Burkholderiales	Methylophilaceae	Methylophilus	Methylophilus_sp000525025(RS_GCF_000525025.1)
Daphnia dentifera	0.000	0.006	Bacteroidota	Bacteroidia	Chitinophagales	Chitinophagaceae	NA	NA
Daphnia dentifera	0.000	0.003	Proteobacteria	Gammaproteobacteria	Burkholderiales	Methylophilaceae	Methylotenera	Methylotenera_mobilis(RS_GCF_000023705.1)
Daphnia dentifera	0.000	0.001	Proteobacteria	Alphaproteobacteria	Sphingomonadales	Sphingomonadaceae	Sphingomonas	NA
Daphnia dentifera	0.000	0.018	Bacteroidota	Bacteroidia	NS11-12g	NA	NA	NA
Daphnia dentifera	0.000	0.158	Proteobacteria	Gammaproteobacteria	Burkholderiales	Burkholderiaceae	Polynucleobacter	Polynucleobacter_difficilis(RS_GCF_003065365.1)
Daphnia dentifera	0.000	0.034	Proteobacteria	Gammaproteobacteria	Burkholderiales	Burkholderiaceae	Hylemonella	Hylemonella_sp001432305(RS_GCF_001432305.1)
Daphnia dentifera	0.000	0.028	Bacteroidota	Bacteroidia	Flavobacteriales	Vicingaceae	BRH-c54	NA
Daphnia dentifera	0.000	0.024	Bacteroidota	Bacteroidia	Flavobacteriales	Crocinitomicaceae	Fluviicola	Fluviicola_taffensis(RS_GCF_000194605.1)
Daphnia dentifera	0.000	0.020	Actinobacteriota	Actinomycetia	Nanopelagicales	Nanopelagicaceae	Planktophila	Planktophila_sp002284895(RS_GCF_002284895.1)
Daphnia dentifera	0.000	0.011	Proteobacteria	Gammaproteobacteria	Burkholderiales	Burkholderiaceae	Acidovorax_D	NA
Daphnia dentifera	0.000	0.009	Actinobacteriota	Actinomycetia	Nanopelagicales	Nanopelagicaceae	Planktophila	Planktophila_sp002284895(RS_GCF_002284895.1)
Daphnia dentifera	0.000	0.008	Proteobacteria	Gammaproteobacteria	Burkholderiales	Burkholderiaceae	Limnohabitans	Limnohabitans_sp003063545(RS_GCF_003063545.1)
Daphnia dentifera	0.000	0.007	Bacteroidota	Bacteroidia	Chitinophagales	Chitinophagaceae	Lacibacter	Lacibacter_luteus(RS_GCF_004118265.1)
Daphnia dentifera	0.000	0.003	Proteobacteria	Gammaproteobacteria	Pseudomonadales	Ketobacteraceae	HdN1	HdN1_sp000198515(RS_GCF_000198515.1)
Daphnia dentifera	0.000	0.003	Verrucomicrobiota	Verrucomicrobiae	Verrucomicrobiales	Akkermansiaceae	Rubritalea	NA
Daphnia dentifera	0.000	0.003	Proteobacteria	Alphaproteobacteria	Rhodobacterales	Rhodobacteraceae	Cypionkella	Cypionkella_psychrotolerans(RS_GCF_001294535.1)
Daphnia dentifera	0.000	0.003	Actinobacteriota	Actinomycetia	Nanopelagicales	Nanopelagicaceae	Planktophila	NA
Daphnia dentifera	0.000	0.003	Actinobacteriota	Acidimicrobiia	Acidimicrobiales	Ilumatobacteraceae	Desertimonas	Desertimonas_flava(RS_GCF_003426815.1)
Daphnia dentifera	0.000	0.002	Proteobacteria	Alphaproteobacteria	Sphingomonadales	Sphingomonadaceae	Novosphingobium	NA
Daphnia dentifera	0.000	0.002	Proteobacteria	Alphaproteobacteria	Rhizobiales	Beijerinckiaceae	Methylobacterium	Methylobacterium_aquaticum_B(RS_GCF_001548015.1)
